# Transcriptional modulation of *AREB-1* by CRISPRa improves plant physiological performance under severe water deficit

**DOI:** 10.1038/s41598-020-72464-y

**Published:** 2020-10-01

**Authors:** Bruno Paes de Melo, Isabela Tristan Lourenço-Tessutti, Joaquin Felipe Roca Paixão, Daniel David Noriega, Maria Cristina Mattar Silva, Janice de Almeida-Engler, Elizabeth Pacheco Batista Fontes, Maria Fatima Grossi-de-Sa

**Affiliations:** 1 Embrapa Genetic Resources and Biotechnology-EMBRAPA CENARGEN, Brasília, DF Brazil; 2 Biochemistry and Molecular Biology, Universidade Federal de Viçosa (UFV), Viçosa, MG Brazil; 3 Medical Biochemistry Institute, Universidade Federal Do Rio de Janeiro (UFRJ), Rio de Janeiro, RJ Brazil; 4 Genomic Sciences and Biotechnology, Universidade Católica de Brasília (UCB), Brasília, DF Brazil; 5 UMR Institut Sophia Agrobiotech INRA/CNRS/UNS, Sophia Antipolis, France; 6 National Institute of Science and Technology in Plant-Pest Interactions (INCTIPP)–BIOAGRO, Viçosa, MG Brazil; 7 National Institute of Science and Technology–INCT PlantStress Biotech–EMBRAPA, Brasília, DF Brazil

**Keywords:** Plant biotechnology, Molecular engineering in plants

## Abstract

Plants are sessile organisms, which are vulnerable to environmental stresses. As such, plants have developed multiple molecular, physiological, and cellular mechanisms to cope with natural stressors. However, these environmental adversities, including drought, are sources of the main agribusiness problems since they interfere with plant growth and productivity. Particularly under water deprivation conditions, the abscisic acid-responsive element-binding protein AREB1/ABF2 plays an important role in drought stress response and physiological adaptation. In this investigation, we provide substantial confirmation for the role of AREB1/ABF2 in plant survival under severe water deficit using the CRISPR activation (CRISPRa) technique to enhance the *AREB1* gene expression. In our strategy, the inactive nuclease dCas9 was fused with an Arabidopsis histone acetyltransferase 1, which improves gene expression by remodeling chromatin. The *AREB1* overexpression promotes an improvement in the physiological performance of the transgenic homozygous plants under drought, which was associated with an increase in chlorophyll content, antioxidant enzyme activity, and soluble sugar accumulation, leading to lower reactive oxygen species accumulation. Finally, we found that the CRISPR-mediated up-regulation of *AREB1* changes the abundance of several downstream ABA-inducible genes, allowing us to report that CRISPRa dCas9-HAT is a valuable biotechnological tool to improve drought stress tolerance through the positive regulation of *AREB1*.

## Introduction

Plants are often exposed to stressful conditions, which trigger signaling networks leading to molecular, cellular and physiological modifications that culminate in stress tolerance. Water deprivation or high salinity induces abscisic acid (ABA) accumulation, which coordinates downstream signaling cascades involved in water-use optimization. A typical symptom of ABA accumulation is the stomatal closure, as the main consequence of an ionic imbalance in guard cells, activating pumps that promote ion efflux and consequent turgor losses^1^. Another typical feature of drought is the imbalance on photosynthetic apparatus that leads to reactive oxygen species (ROS) production and accumulation^2^. ROS can act as signaling molecules that regulates several stress-associated processes, encompassing other protective mechanisms, such as the production and accumulation of osmolytes, electron carriers and improvement of transcription, translation, and activity of antioxidant enzymes, among others^1–3^. Collectively, these mechanisms comprise a sophisticated and intricate hormone-responsive pathway, whose transcription factors appear as nodes on signal integration and gene expression remodeling. Desiccating regulatory gene networks and the role of transcription factors in global gene expression changes during the stress provide an effective and useful tool for biotechnological intervention and modern crop breeding programs.

Plants display a large set of transcription factor families involved in stress responses, such as NACs, bZIPs, and WRKYs^4–6^. In the ABA-dependent signaling pathway, one of the most important and well-characterized drought responses is assembled via the basic leucine zipper (bZIP) transcription factor AREB-1 (ABA-responsive element-binding protein 1), which emerges as a central regulator of drought-inducible adaptive pathways^5^.

The AREB transcription factors can recognize a widely conserved sequence in promoters of different stress-responsive genes. These elements, named ABREs (ABA-responsive elements), are constituted by the PyACGTGG/TC sequence^7–9^, and a monohybrid approach in Arabidopsis has identified nine AREB family members with possible overlapping functions^10–12^. In Arabidopsis, AREB-1, AREB-2, and AREB-3 cooperatively regulate the ABA-dependent signaling pathway. The triple mutant plants display an extremely low survival ratio when submitted to severe dehydration followed by rehydration and higher water and biomass losses than WT and single-mutant plants under drought stress^12,13^. In addition, the overexpression of *AREB-1* under the *CaMV 35S* constitutive promoter has been demonstrated to be a good strategy for drought tolerance improvement in different plant species, such as Arabidopsis, rice, and soybean^14–17^.

Recently, our research group demonstrated that positive transcriptional modulation regulation of *AREB-1* using dCas9-HAT (histone acetyl-transferase) leads to constitutive expression of *AREB-1* and *RD29A*, other downstream genes of the AREB pathway^16^, in transgenic *Arabidopsis thaliana* plants by CRISPRa. The CRISPRa approach constitutes a powerful biotechnological tool for stress tolerance improvement in plants. The gene overexpression approach using dCas9-HAT can be considered superior to the classical strategy using *CaMV 35S* because we can directly target specific cis-acting elements in gene promoters through the sgRNA use, avoiding the pleiotropic effects and post-transcriptional gene silencing despite the use of constitutive viral promoters.

The overexpression of *AREB* genes has been shown to provide significant improvements in osmotic stress tolerance, whereas the knock-down or knock-out mutants showed elevated sensitivity to osmotic stress. Nevertheless, in some cases, the overexpression of these genes leads to negative effects, which comprise reduced productivity, delay in development and growth. A considerable number of commodities have limited production due to water availability^13,14,16^.

We previously provided a functional characterization of homozygous Arabidopsis lines, in which *AREB-1* is upregulated by the CRISPRa approach^16^. The AREB1-OX lines display a significant increase in *AREB-*1 expression, reaching at least twofold as compared to untransformed plants. Either during severe drought stress or mild-severe drought stress, the transgenic plants display enhanced physiological performance compared to that of the WT control. The chlorophyll loss ratio and stomatal opening in AREB1-OX plants were lower than those observed in WT plants, suggesting superior stress-associated physiological performance during drought. In addition, the survival ratio corroborates these data: after re-watering, AREB1-OX plants were almost totally recovered, whereas only 50% of WT plants did^16^.

Hereafter, we provide a complete molecular, biochemical, and physiological characterization of a transgenic line of *A. thaliana* overexpressing *AREB-1* (AREB1-OE) simulating closed field drought conditions. Our analyses reinforce the applicability of genetic engineering in modern plant breeding programs to achieve agronomic relevant traits in different crops, according to the demands of each culture.

## Results and discussion

### CRISPRa plants overexpressing AREB-1 display classical phenotypes of drought tolerance

**Figure 1:**
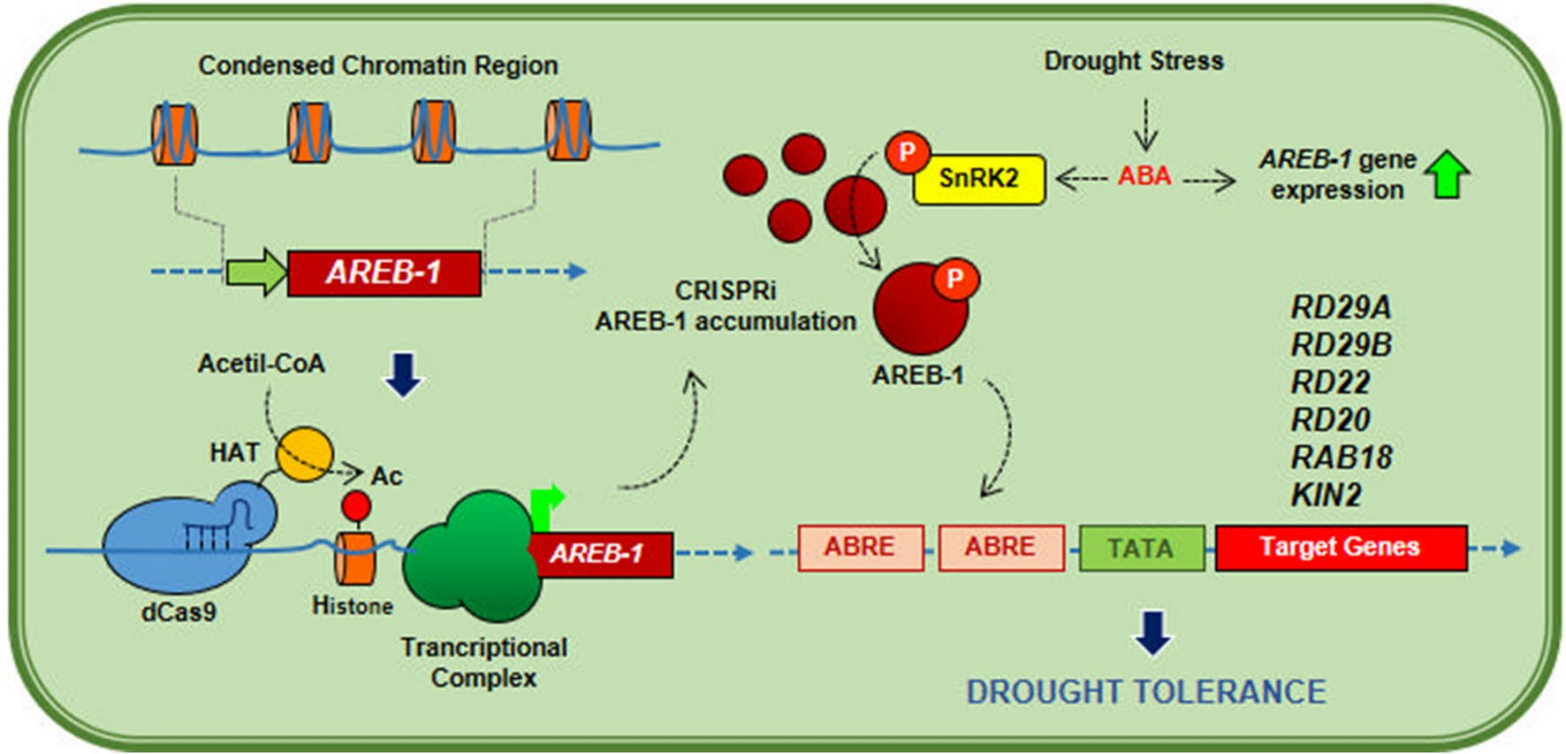
Schematic representation of the strategy of CRISPRa-mediated *AREB-1* overexpression in drought-tolerance improvement. A sgRNA targeting the *AREB-1* promoter region drives nuclease-inactive dCas9 fusion with *A. thaliana* histone acetyl-transferase. Histone acetylation causes structural changes in chromatin and facilitates the transcriptional machinery assembly, resulting in enhanced gene transcription and consequent protein accumulation. The accumulation of AREB-1 acts as a drought-preventive mechanism: during drought, plant cells release ABA, which activates the transcription of *AREB-1* and its transcriptional activity. Because of AREB-1 accumulation, ABA accumulation during the early stages of drought allows for its full activation, and triggers the transcriptional activation of several mechanisms of many genes involved in ABA-mediated drought tolerance, culminating in morpho-physiological changes, which enhances the CRISPRa plants’ performance under severe water deprivation.

**Figure 2:**
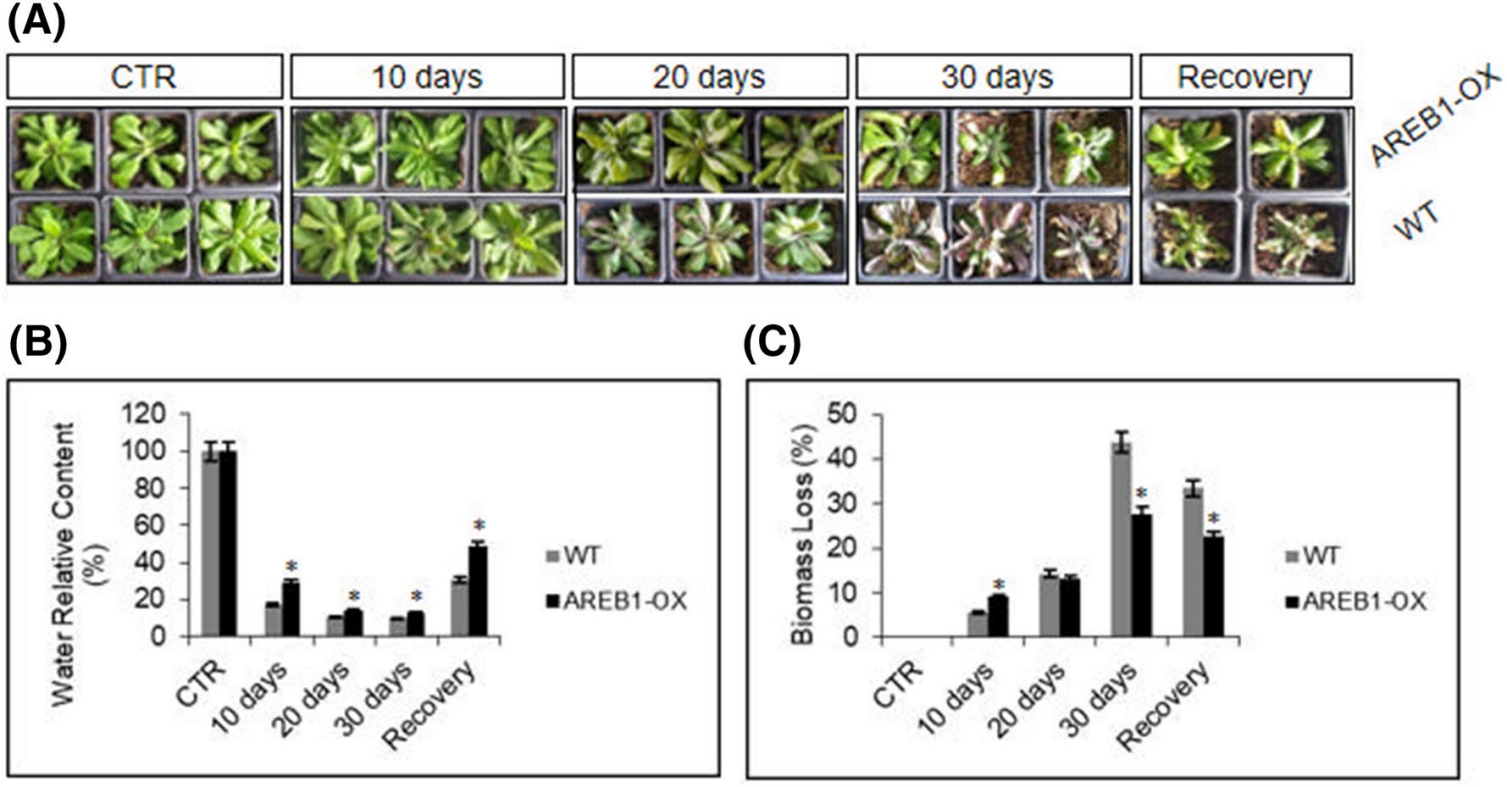
The overexpression of *AREB-1* leads to a classical drought-tolerance phenotype under severe water deprivation. To simulate a closed field drought, 5-week-old *A. thaliana* WT and homozygous lines overexpressing *AREB-1* were exposed to water deprivation for 30 days. (**A**) During drought, the transgenic lines display the classical phenotype of drought tolerance, exhibiting a low ratio of leaf yellowing and curliness, reinforced by the green, healthy phenotype of plants. The WT plants displayed, in addition to leaf yellowing, the production of defensive metabolites triggered by severe drought stress, indicated by purple-colored leaves. After recovery, AREB1-OX plants were almost fully recovered and only 45–50% of the WT plants recovered. (**B**) Relative water content (RWC) during drought. All pots were filled with 98 g of moist soil and *watered normally until drought* stress. During water deprivation, the pots were weighed, and the RWC was calculated according to the initial weight. The RWC values were normalized to the value at 100% RWC of the control groups. (**C**) Biomass loss ratio. The regularly watered plants were randomly distributed on trays and analyzed at intervals of 10 days under 30 days of water deprivation. After drought stress, plants were re-watered for 1 week to calculate the plant survival ratio. Control plants were normally watered during the stress period, and biomass loss was set as 0%. Values represent the increasing biomass loss as compared to 0% of the control group. All analyses were conducted with 3 biological and 2 technical replicates composed of a pool of 9 plants. The bars indicate 5% standard error, and asterisks indicate t-test statistical significance under 95% confidence.

The transcriptional modulation of *AREB-1* expression by CRISPR was capable of upregulating many important genes related to drought adaptability through the ABA-dependent signaling pathway in plants. *AREB-1* expression improved their physiological performance under severe water deprivation (Fig. 1). To investigate whether AREB1-OX plants displayed enhanced physiological performance during drought, 5-week-old transgenic and WT plants were submitted to gradual desiccation by plant watering suspension for 30 days, followed by re-watering for 1 week (Fig. 2). During the stress, the AREB1-OX plants exhibited a high survival ratio, with a green and healthy phenotype even at 30 days of water suspension (Fig. 2A). After 10 days of stress, the differences between the WT and AREB1-OX plants were not significant. However, after 20 days, the effect of AREB-1 accumulation was notable, giving the transgenic plants more robustness than the WT plants, which displayed curly, dehydrated, and purple- and yellow-colored leaves (Fig. 2A). These symptoms indicated high levels of ROS and secondary metabolite production in response to drought. The enhanced performance of the AREB1-OX plants during stress allowed for complete recovery after re-watering.

We also investigated the drought tolerance of transgenic plants by analyzing the relative water content (RWC) and biomass loss under water scarcity conditions. The RWC observed in transgenic plants was superior to that in WT plants during drought (Fig. 2B). Even 10 days after water deprivation, when AREB1-OX and WT plants did not exhibit contrasting phenotypes, the RWC was at least two fold higher in the transgenic lineages than in the WT plants, demonstrating that the overexpression of *AREB-1* sustained the plants under drought conditions and enhanced adaptive physiological mechanisms during mild-severe stress. These responses could be associated with the most efficient water use ratio or morphological changes that allowed the plants to have enhanced water uptake and/or better evapotranspiration balance.

More importantly, drought-tolerant plants displayed low biomass losses. The biomass reduction rates of CRISPRa AREB-OX plants were significantly lower than the ratios observed in WT plants (Fig. 2C) at a similarly reduced soil moisture content. The biomass loss of AREB1-OX plants was almost the same after 10 and 20 days of stress. In contrast, the WT plants displayed a two fold increase in biomass loss during the same period. The RWC and biomass loss ratios are associated with water use efficiency and evapotranspiration balance in plants.

### The overexpression of AREB-1 promotes a protective effect against drought-induced oxidative stress

As a result of water deprivation, plants under drought display imbalanced photosynthesis, and high levels of oxidative damage caused by ROS accumulation^18,19^. Since the transgenic plants showed a lower degree of leaf damage than the WT plants, we investigated whether CRISPRa AREB-OX plants also display lower ROS accumulation and consequent lower cell membrane damage.

**Figure 3:**
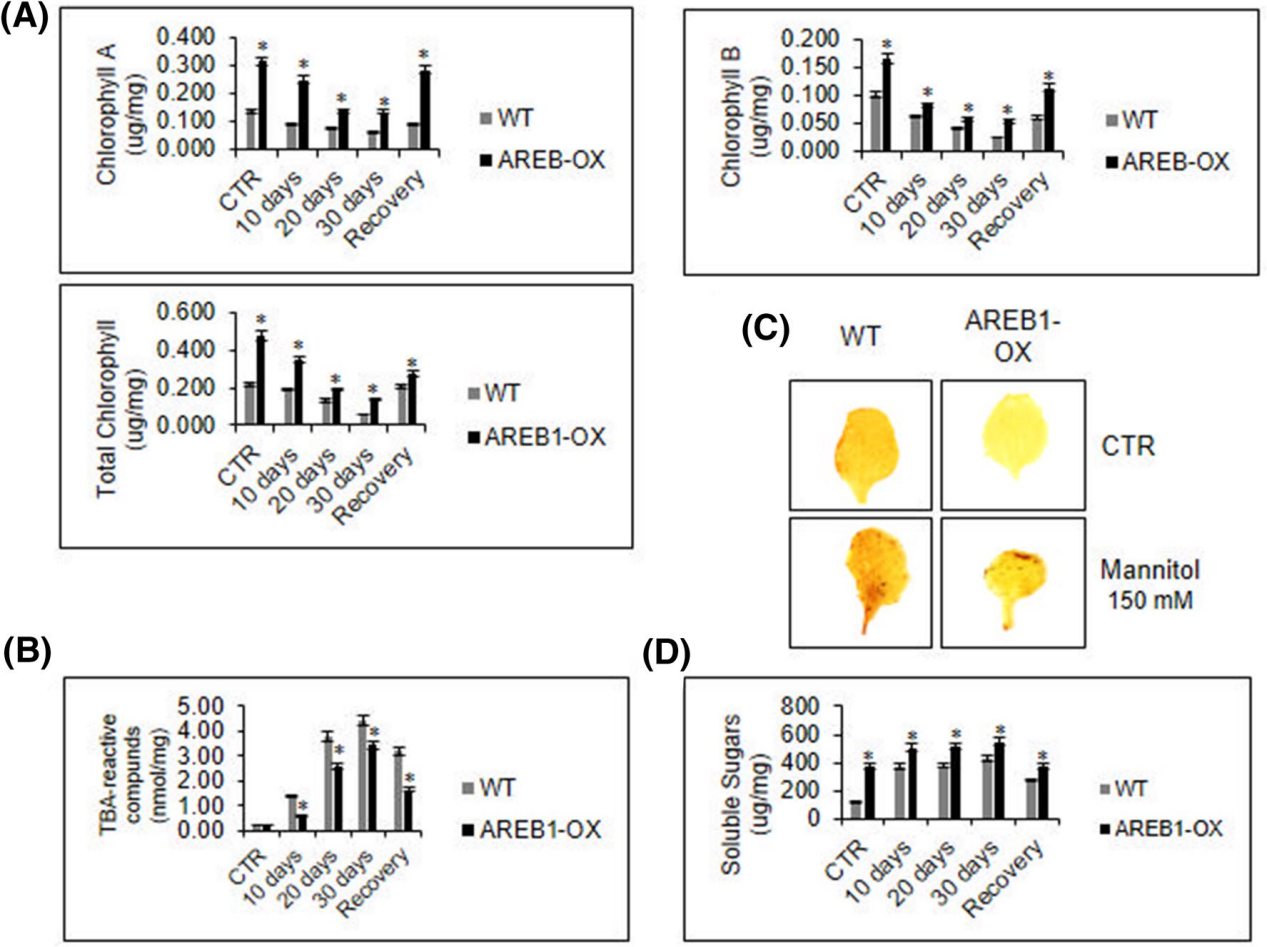
Biochemical characterization of AREB1-OX plants under drought. (**A**) Chlorophyll levels. The levels of chlorophyll A, B, and total chlorophyll were determined in ethanolic extracts during water deprivation by spectrophotometry at 645 nm and 663 nm. Total chlorophyll was expressed as µg/mg of tissue. (**B**) TBA-reactive compounds. In several stresses, plant cells produce ROS. During drought, the collapse of the photosynthetic apparatus enhances the effects of tissue oxidation, culminating in higher levels of malondialdehyde, a TBA-reactive compound produced during lipid peroxidation. (**C**) DAB-mediated hydrogen peroxide detection in leaves. Stressed leaves were stained with DAB and distained in an ethanol bath for H_2_O_2_ detection. (**D**) Soluble sugar quantification. The soluble sugars were spectrophotometrically quantified in ethanolic extracts by the DNS method using a glucose-based standard curve. Biochemical analyses were conducted with 3 biological and 2 technical replicates. The bars indicate 5% standard error, and asterisks indicate t-test statistical significance under 95% confidence.

Our results demonstrated that under 30 days of water deprivation conditions, the transgenic plants displayed higher levels of chlorophyll A than the WT plants (Fig. 3A). The levels of chlorophyll B were also significantly higher in transgenic lines than in WT plants (Fig. 3A). After recovery, the transgenic plants exhibited levels of chlorophyll close to those displayed by control plants, indicating an appreciable recovery capacity that was not displayed by the wild-type plants (Fig. 3A). Finally, the levels of total chlorophyll in AREB1-OX plants are higher than in WT as an adaptive preset mechanism, independently on the condition, which justifies the green and healthy phenotype previously observed as an effect of *AREB-1* up-regulation in transgenic plants.

We also compared the levels of H_2_O_2_ and TBA-reactive compounds, which are reliable biomarkers of drought-induced oxidative stress and lipid peroxidation, respectively. Under well-watered conditions, the content of TBA-reactive compound, which includes malonaldehyde (MDA), did not significantly differ between WT and AREB1-OX plants (Fig. 3B). During dehydration, imbalances in the photosystem lead to the formation of H_2_O_2_, whose damage can be observed in several varieties of biological molecules, such as membrane lipids^18,20,21^. Drought stress enhanced the accumulation of the TBA-reactive compound MDA in the WT and AREB1-OX plants, although to a different extent. The MDA levels were remarkably higher in WT plants than in transgenic plants. In the WT plants, the MDA levels increased 2.5- to 3.0-fold after 10 days and 8.0-fold after 20 days of treatment compared to those of the well-watered plants (Fig. 3B). In AREB1-OX plants the drought-mediated increases in TBA-reactive compounds were as low as 0.5- to 1.0-fold and 4.0- to 5.0-fold after 10 and 20 days of treatment, respectively, suggesting an enhanced antioxidant system in the transgenic plants.

As expected, the levels of H_2_O_2_ were also higher in WT plants than in transgenic plants submitted to a hyperosmotic environment. The accumulation of H_2_O_2_ was lower in transgenic plants, as indicated by the light-brown DAB-stained leaves (Fig. 3C). These results were closely linked to the lower MDA yield in transgenic plants and the phenotypical characterization results. The enhanced capacity to uptake water and the fine-tuned controlled evapotranspiration did not allow for electrons to escape in the photosystems, inhibiting the production of ROS and reducing the consequent chlorophyll losses and MDA production during drought. Associated with the higher RWC and chlorophyll content during severe or mild-severe stress as compared to the WT plants, these results confirm that the overexpression of *AREB-1* improves the mechanisms that preserve the cell water content, mitigating oxidative damage during drought.

**Figure 4:**
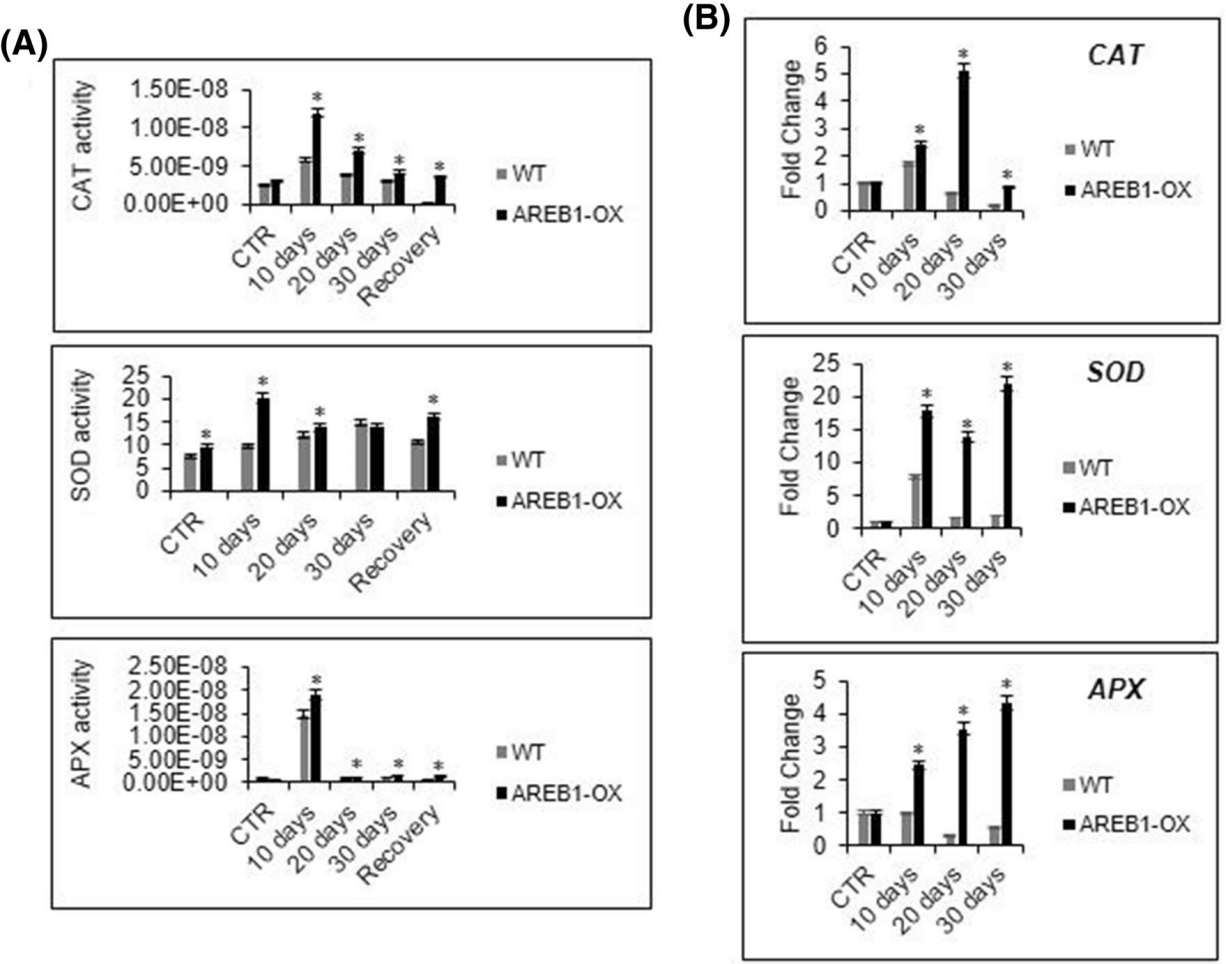
Antioxidant enzymatic activity. (**A**) Enzymatic activity of superoxide dismutase (SOD), catalase (CAT) and ascorbate peroxidase (APX) during drought. The enzymatic activity was spectrophotometrically determined in the whole protein extract, and total protein was determined by the Bradford method. SOD specific activity was expressed as U/total protein, and CAT and APX specific activities were expressed as µmol H_2_O_2_/min/mg protein and µmol ascorbate/min/mg protein, respectively. All analyses were conducted with 3 biological and 3 technical replicates. The bars indicate 5% standard error, and asterisks indicate *t* test statistical significance under 95% confidence. (**B**) Antioxidant enzyme transcript accumulation. The transcript accumulation of the enzyme genes was quantified by qRT-PCR. Fold change (FC) was calculated by the 2^−ΔΔCt^ method, and the results are expressed in a CTR-normalized manner. Gene expression analysis was conducted with 3 biological and 2 technical replicates. The bars indicate 5% standard error, and asterisks indicate *t* test statistical significance under 95% confidence.

To determine whether the decreased MDA and H_2_O_2_ levels in transgenic plants resulted from some mechanism of cell-water maintenance and increased antioxidant activity, we determined the content of soluble sugars and the activity of SOD, CAT, and APX antioxidant enzymes (Figs. 3D, 4). One of the mechanisms for avoiding water loss during drought is the enhancement of osmolyte synthesis. The accumulation of simple sugars, such as fructose and glucose, or amino acids, such as proline, reestablishes the osmotic balance, maintaining cell homeostasis^5^. The WT and transgenic lineages accumulated, stably, soluble sugars during stress, exhibiting small ratio variance over time. The transgenic lineages displayed higher sugar content even in well-watered conditions, as observed for the chlorophyll content. These results may suggest that the overexpression of *AREB-1* could affect the regulation of osmotic control mechanisms in plant cells (Fig. 3D).

Under normal water availability, SOD, but not CAT and APX, displayed significantly higher activity in the AREB1-OX plants, which may be a result of CRISPRa-mediated up-regulation of *AREB-1* (Fig. 4A) In contrast, the activities of all three examined enzymes were higher in both transgenic and WT plants under drought, although to a greater extent in AREB1-OX plants (Fig. 4A). Consistent with this result, the expression levels of SOD, CAT and APX genes were higher in AREB1-OX than in WT plants under drought (Fig. 4B).

In well-watered control plants, the levels of TBA-reactive compounds and antioxidative enzyme activities (except for SOD) were not correspondingly different between the WT and transgenic plants (Figs. 3B, 4A). AREB-1 transcription and protein accumulation on their own cannot promote notable improvements in plant physiology because ABA, which is required to activate AREB-1 fully, is produced mainly under drought. Collectively, these mechanisms indicate that the constitutive expression of AREB-1 may act as a preset mechanism of drought response and that previous accumulation of AREB-1 allows for efficient and quick activation in the presence of ABA. AREB-1 positively regulates the mechanisms involved in the antioxidative protection, providing CRISPRa *AREB-1-*enhanced plants with an improved antioxidant system, which mainly contributes to protection against oxidative damage and consequent cell death triggered by drought stress.

### Downstream genes in the ABA-mediated drought-responsive stress pathway are upregulated in transgenic plants overexpressing AREB-1

**Figure 5:**
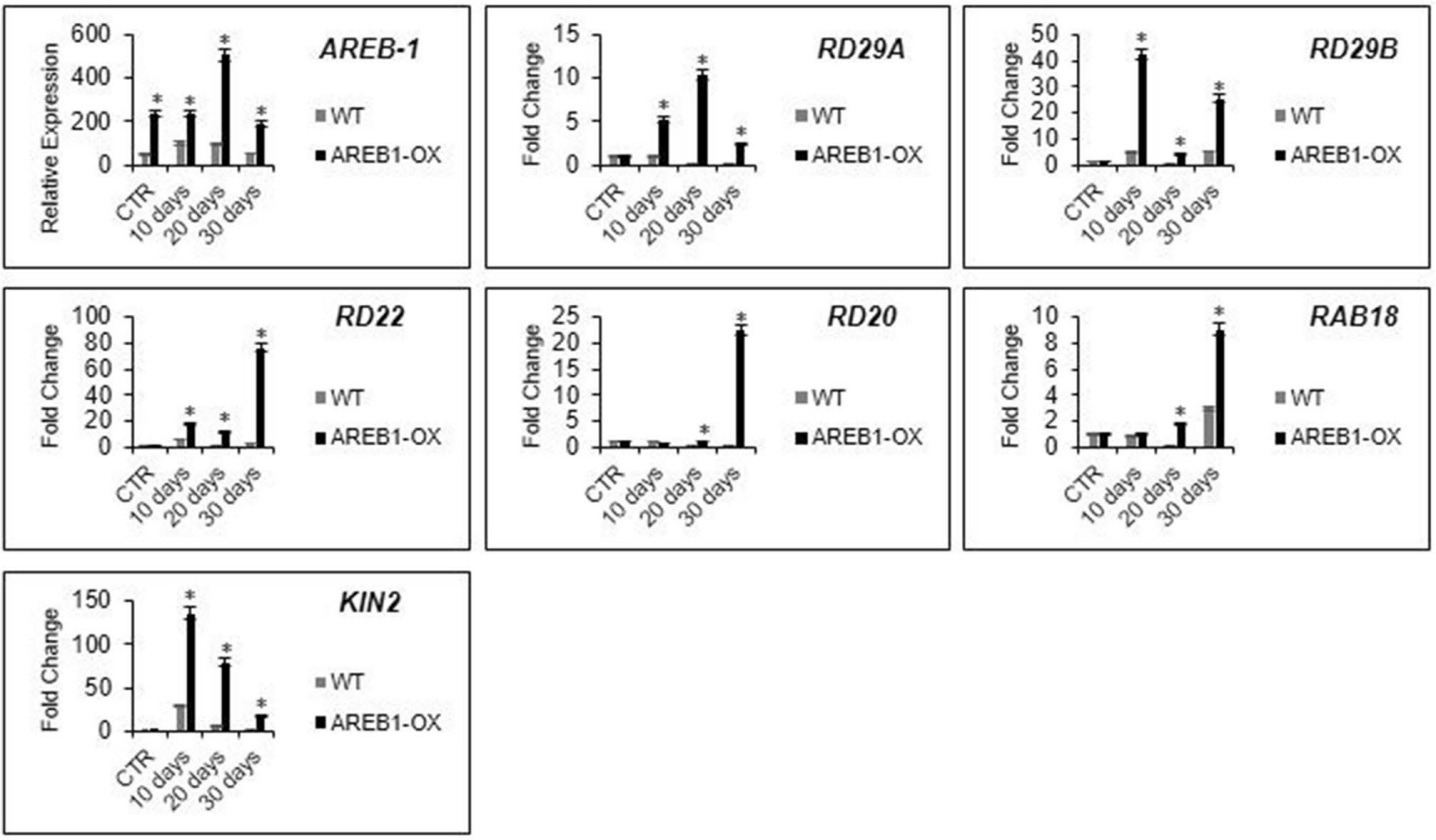
The effect of *AREB-1* overexpression on transcriptional regulation of ABA-responsive downstream genes. The water deprivation increases ABA content in plant cells, which triggers an adaptive osmotic stress response via morpho-physiological changes. High levels of ABA up-regulate the expression of AREB-1, which is fully activated by SnRK2-mediated phosphorylation, which isalso positively regulated by ABA. Once active, AREB-1 can bind ABRE *cis*-elements on ABA-responsive promoters, eliciting a drought-responsive pathway that confers tolerance. The level of expression of *AREB-1* was determined at each predetermined time point during water deprivation. As expected, the relative expression was markedly higher in the CRISPRa lines than in the WT plants. The relative expression was calculated using the 2^−ΔCt^ method and expressed as a relation between endogenous and target-gene transcript levels. The expression of the *AREB-1* downstream genes (*RD29A*, *RD29B*, *RD22*, *RD20*, *RAB18,* and *KIN2*) was determined by the 2^−ΔΔCt^ method, and FC was expressed in a CTR-normalized manner. Gene expression analysis was conducted with 3 biological and 2 technical replicates. The bars indicate 5% standard error, and asterisks indicate *t* test statistical significance under 95% confidence.

ABA-responsive genes display multiple ABREs or a combination of them with other complementary elements that enhance the transcription of these genes under drought or ABA accumulation^13,22–24^. Given that overexpression of *AREB-1* in Arabidopsis led to notably enhanced drought tolerance, we investigated whether the phenotypic and physiological changes in transgenic plants were correlated with the changes in the expression of downstream genes in the ABA-mediated pathway. These genes included *RD29A*, *RD29B*, *RD22* and *RD20*, *RAB18,* and *KIN2* (Fig. 5).

AREB-1 controls the expression of two classes of drought-responsible genes: (a) LEA genes, which encompass a large group of encoded proteins that display LEA or LEA-like hydrophilic proteins and accumulate during embryogenesis and dehydration and (b) regulatory genes involved in ABA signal transduction and gene expression regulation^14^.

Among our analyzed stress markers, RAB 18 is a classical dehydrin of the group 2 LEA proteins, and RD29B and KIN2 are LEA-like proteins, as they do not display canonical consensus motifs but have relatively high amino acid similarity with group 1 and group 2^14^. In addition, the promoter of *RD29B* displays two ABRE sequences, and its expression is strongly related to drought responses mediated by ABA induction^25^.

As expected, the overexpression of *AREB-1* upregulated the genes carrying ABRE sequences. During drought, the expression levels of the marker genes demonstrated variable kinetics, but these levels were always higher in the transgenic plants than in the wild type plants (Fig. 5). This variable pattern is already described for other stress- responsive transcription factors, such as NAC transcription factors under multiple stresses in plants^4^. During the stress, the transcription factors that belong to hormone-controlled stress-responsive pathways have their expression modulated by a multilayered network of signal transducers according to the environmental and the intracellular signals. The expression of upstream genes in ABA-responsive pathways, such as *KIN2*, decreases along time after activating downstream cascades. Multiple physiological features lead to a variable expression of *RD29A, RD29B, RD22, RD20* and *RAB18* while plants try to cope with longstanding adverse conditions upon activation of programmed cell death pathways in severe stresses^5–7,12^. In well-watered plants, except for *AREB-1* expression, the levels of the marker genes were not different probably because AREB-1 was not fully activated in the absence of ABA.

## Conclusion

In conclusion, our study shows that overexpression of *AREB-1* mediated by the CRISPRa strategy results in highly improved drought stress responses, encompassing a powerful biotechnology tool for genetic engineering. We provide a complete physiological and biochemical characterization of the AREB-1 function in drought tolerance, reinforcing the relevant role of ABA-related transcription factors in the activation of several molecular mechanisms of stress coping. Collectively, the data demonstrate remarkable transcriptional activation of genes involved in drought stress tolerance, culminating in the improvement of morphological and molecular characteristics related to enhanced plant performance under water deprivation regimes (Fig. 5). The transcriptional activation of *AREB-1* leads to lower biomass loss and higher relative water content during 30 days under water deprivation conditions than those of the WT plants. The water deficit tolerant phenotype may be associated with higher antioxidant enzyme activity, and higher levels of soluble sugars, which regulated the efficiency of water use and consequent ROS accumulation, chlorophyll maintenance, and plant survival.

## Methods

### Plant growth and stress treatment

The transgenic homozygous Arabidopsis seeds of AREB1-OX and WT ecotype Columbia (Col 0) were germinated on soil under controlled growth-chamber conditions: 22 °C, a 12 h/12 h photoperiod and 70% humidity. After 2 weeks, the seedlings were transferred to individual pots filled with 98 g of moist soil homogenized with glufosinate-ammonium (100 mg/L). The pots were randomly distributed in trays, exposed to the same light intensity and regularly watered with 500 mL of water two times/week. The controlled conditions in the growth chamber and experimental design should be precisely followed to guarantee similar reducing rate in the soil moisture to all lines during the drought assay. After acclimation, all pots were weighed, and the watering was suspended for 30 days to generate osmotic stress. The control group (unstressed plants) consisted of plants under regular and controlled watering. The samples for physiological assays were collected every 10 days after water deprivation and immediately frozen in liquid nitrogen. The pots were weighed during each sample collection. After 30 days under continuous drought, the plants were irrigated daily with 200 mL of water for 1 week. All analyses were conducted with 3 biological and 2 technical replicates composed of a pool of 9 plants.

### RNA extraction and cDNA synthesis

Total RNA was extracted from 100 to 150 mg of leaves using TRIzol (Invitrogen, Carlsbad, CA-USA) according to the manufacturer’s recommendations. The RNA was quantified spectrophotometrically using a NanoDrop instrument (Thermo Fisher, Waltham, Massachusetts, USA), and its quality and integrity were evaluated by electrophoresis on a 1% (w/v) agarose gel. A total of 1 µg of RNA was used to perform cDNA synthesis according to the MMLV reverse transcriptase (Invitrogen, Carlsbad, CA-USA) protocol.

### qPCR and gene expression analysis

The expression profile of ABA-related and antioxidant enzyme genes was performed with a QuantStudio3 qPCR System (Thermo Fisher, Waltham, Massachusetts-USA) using SYBR Green (Invitrogen, Carlsbad, CA-USA) reagent according to the manufacturer’s recommendations. All genes were analyzed with 3 biological samples and 2 technical replicates using gene-specific primers (Supplementary Table 1). The reaction was performed as follows: 2 min at 50 °C, 10 min at 95 °C, and 40 cycles of 94 °C for 15 s and 60 °C for 1 min. *GAPDH* and *EFL1A* were chosen as endogenous control genes (normalizer), and relative gene expression was calculated by the 2^−ΔΔCt^ comparative method.

### Relative water content and biomass loss

During the stress treatment, the pots were filled with the same amount of soil moisture and watered with the same volume of water. After the seedling transfer, the pots were well-watered for 1 week, and their initial weights were recorded. During water deprivation, the pots were weighed every day for 10 days. To calculate the relative water content (RWC), the weight of each pot was compared with the weight of the well-watered pot, and the difference between them was expressed as RWC (%) according to the following equation: [RWC = (100 × W_n_)/W_0_], in which W_n_ represents the weight at the time of analysis (expressed in days under water deprivation; n = 10, 20 or 30). W_0_ represents the weight of the well-watered pot. For recovered plants, the same equation was used replacing W_n_ by W_r_, in which W_r_ = weight of re-watered pot.

To calculate biomass loss (BL), the initial weight of each plant was considered to be 1, and the biomass loss was expressed as a percentage throughout the water deprivation period, comparing the weight of the plant at the time of analysis and that of the well-watered plant at the beginning of the stress period. The equation used in this analysis was [BL = ((W_0_–W_n_) × 100)/W_0_], in which W_n_ represents the plant weight at the time of analysis and W_0_ represents the weight of the well-watered plant.

### Chlorophyll content

Total chlorophyll was determined spectrophotometrically, according to described by Melo et al.^4^. Approximately 100 mg of leaves were weighed, and total chlorophyll was extracted with 1 mL of absolute ethanol in a dark tube. The ethanolic extract was centrifuged at 12,000*g* and 4 °C for 15 min, and the supernatant was quantified in a spectrophotometer at 645 nm and 663 nm. Total chlorophyll was expressed in µg/mg of tissue.

### Sugar content

The reducing sugar content was determined in the ethanolic extract by the 3,5-dinitrosalycilic acid (DNS) method. DNS (0.1% w/v) was prepared in sodium and potassium tartrate (30% w/v) and sodium hydroxide (0.4 M). An aliquot of 100 µL of leaf extract was added to 900 µL of water and 500 µL of DNA, and the reaction was performed in a boiling water bath for 5 min. After the reaction, the samples were immersed on ice, and their absorbance was acquired with a spectrophotometer at 540 nm. The sugar content was determined using a glucose-based standard curve (0–1.2 µg/µL).

### Oxygen species analysis

Reactive oxygen species accumulation was analyzed by quantification of TBA-reactive compounds and in situ H_2_O_2_ staining. Lipid peroxidation is a process with a final product of malondialdehyde (MDA), which reacts with thiobarbituric acid (TBA). TBA-reactive compounds were quantified as described by Melo et al.^4^. Approximately 200 mg of leaves were homogenized in 2 mL of 0.1% (v/v) trichloroacetic acid (TCA) and then centrifuged at 12,000*g* for 15 min. An aliquot of 500 µL of the clarified supernatant was added to 1.5 mL of 0.5% (w/v) thiobarbituric acid (TBA) in 20% (v/v) TCA, and the samples were incubated at 90 °C for 20 min. The reaction was stopped by incubation on ice, followed by centrifugation for 4 min. The absorbance of the supernatant was measured at 532 nm, and the concentration of TBA-reactive compounds was calculated considering the MDA molar extinction coefficient of 155 mM cm^−1^.

For hydrogen peroxide staining, the plants were submerged in a mannitol (150 mM) solution for 24 h, and the leaves were carefully removed and immediately incubated with 3,3-diaminobenzamidine (DAB) solution (1 mg/mL, pH 3.8) for 8 h under continuous agitation. After staining, the leaves were distained in 100% ethanol until total chlorophyll was lost. The leaves were rehydrated in water and glycerol (10% v/v) and photographed stereoscopically.

### Antioxidant enzyme activity

The enzymatic activities of superoxide dismutase (SOD), catalase (CAT) and ascorbate peroxidase (APX) were determined in the whole protein extract. The proteins from 150 to 200 mg of leaves were extracted in 1 mL of 100 mM phosphate buffer pH (7.8) and added to DTT (0.5 g/L), EDTA (10 µM) and PVPP (0,3 mg/mL). Then, all samples were centrifuged at 12,000*g* and 4 °C for 15 min. An aliquot of 500 µL of the clarified supernatant was transferred to 500 µL of extraction buffer and kept on ice until enzymatic activity determination. All enzymatic activities were determined in 3 biological samples and 2 technical replicates, and total protein was determined by the Bradford method.

For SOD activity, a reaction mix was prepared with 100 µL of phosphate buffer, 40 µL of methionine solution (12 g/L), 2 µL of EDTA (3.5 g/L), 15 µL of NBT (0.35 g/L), 2 µL of riboflavin (0.008 g/L), 31 µL of milli-Q water, and 10 µL of protein extract for each reaction. An aliquot of 200 µL of the reaction mix was separated to be used as the “absolute blank”, and another aliquot was separated to be used as the “light blank” during NBT light-mediated reduction. The assay was performed in chilled 96-well microplates avoiding exposure of the samples to light. NBT reduction was performed under intense white light exposure for 40 min, and the absorbance was immediately measured in a spectrophotometer at 560 nm. SOD activity was expressed in relation to the percentage of inhibition of light-mediated NBT reduction. To calculate SOD activity, the inhibition percentage was calculated by the expression IN (%) = $\frac{{{\text{LB}} - {\text{SA}} - {\text{AB}}}}{{{\text{LB}}}}$, in which LB = light blank, SA = sample absorbance, and AB = absolute blank. Once the IN (%) was calculated, it was used to calculate the units of SOD (U), considering that 1 SOD unit is necessary to inhibit 50% of light-mediated NBT reduction. Finally, the specific SOD activity was calculated by the expression SOD = $\frac{{{\text{IN }}\left( {\text{\% }} \right){ }.{ }10}}{{{\text{U }}.{\text{ TP}}}}$, in which IN (%) = the percentage of inhibition, U = SOD units, and TP = total protein (µg/g). The SOD specific activity was expressed in U/total protein.

The CAT activity was determined using a reaction mixture composed of 144 µL of phosphate buffer, 30 µL of water and 16 µL of hydrogen peroxide solution (50 mM). To determine CAT activity, the reaction mixture was added to 10 µL of protein extract, and the absorbance reading was performed for 10 min at intervals of 30 s at 240 nm using a chilled optical 96-well plate. To calculate CAT activity, the absorbance of samples was subtracted from the blank absorbance by the expression CAT = $\frac{{{\text{dA }}.{ }0,0067}}{{{\text{T }}.{ }39,4{ }.{\text{ W }}}}$, in which dA = (initial absorbance–final absorbance), T = time of reaction, 39.4 mol cm^−1^ represents the molar extinction coefficient of hydrogen peroxide, and W = sample weight. The CAT specific activity is expressed in µmol H_2_O_2_/min/mg protein.

The APX activity was determined as described for CAT, and the reaction mixture was added to 10% (v/v) ascorbic acid solution (18 g/L). The molar extinction coefficient of ascorbic acid is 2.8 mol cm^−1^, and the absorbance was measured at 290 nm. As for CAT activity, APX activity is expressed in µmol ascorbate/min/mg protein.

## Supplementary information

Supplementary Table S1.
