# Supplementary material for: Transcriptional modulation of AREB-1 by CRISPRa improves plant physiological performance under severe water deficit
Source: Sci Rep. 2020 Oct 1;10:16231. doi: 10.1038/s41598-020-72464-y (PMC7530729; doi:10.1038/s41598-020-72464-y)
Supplement: Supplementary file 1 — Supplementary Table S1. [file 41598_2020_72464_MOESM1_ESM.docx]

**Transcriptional modulation of *AREB-1* by CRISPRa improves plant physiological performance under severe water deficit**

Bruno Paes de Melo^1,2, *^, Isabela Tristan Lourenço-Tessutti^1*^, Joaquin Felipe Roca Paixão^1,3^, Daniel David Noriega^1,4^, Maria Cristina Mattar Silva^1^, Janice de Almeida-Engler^5^, Elizabeth Pacheco Batista Fontes^2,6^, Maria Fatima Grossi-de-Sa^1,5.7, δ^

* These authors contributed equally to this work

^1^ Embrapa Genetic Resources and Biotechnology-EMBRAPA CENARGEN, Brasilia-DF, Brazil

^2^ Federal University of Viçosa (UFV)-Biochemistry and Molecular Biology, Viçosa-MG, Brazil

^3^ Federal University of Rio de Janeiro (UFRJ)-Medical Biochemistry Institute, RJ, Brazil

^4^ Catholic University of Brasília (UCB)-Genomic Sciences and Biotechnology, Brasília-DF, Brazil

^5^ UMR Institut Sophia Agrobiotech INRA/CNRS/UNS, Sophia Antipolis, France

^6^National Institute of Science and Technology in Plant-Pest Interactions (INCTIPP)–BIOAGRO, Brazil

^7^ National Institute of Science and Technology–INCT PlantStress Biotech–EMBRAPA, BRAZIL

**Email addresses of all authors:**

Bruno Paes de Melo: [brunopaesdemelo@gmail.com](mailto:brunopaesdemelo@gmail.com)

Isabela Tristan Lourenço-Tessutti: [isabelatl@gmail.com](mailto:isabelatl@gmail.com)

Joaquin Felipe Roca Paixão: joaquinfrp@gmail.com

Daniel David Noriega: daniel.nv07@gmail.com

Maria Cristina Mattar da Silva: cristina.mattar@embrapa.br

Janice de Almeida-Engler: janice.de-almeida@inrae.fr

Elizabeth Pacheco Batista Fontes: bbfontes@ufv.br

Maria Fatima Grossi-de-Sa: (^δ^ Corresponding author) [fatima.grossi@embrapa.br](mailto:fatima.grossi@embrapa.br) +556134484705

**Supplementary data**

**Supplementary Table S1.** Primers were used in this study

| **Purpose** | **Name** | **Sequence** |
| --- | --- | --- |
| Endogenous control gene in *Arabidopsis* | ACT2-F | 5'-GATCTCCAAGGCCGAGTATGAT-3' |
|  | ACT2-R | 5'-CCCATTCATAAAACCCCAGC-3' |
| *AREB-1*  Downstream genes | CAT-F | 5'-TGGGATTCAGACAGGCAAGAACG-3' |
|  | CAT-R | 5'-GTTTGGCCTCACGTTAAGACGAGT-3' |
|  | SOD-F | 5'-TGAACTCAGCCTGGCTACTGG-3' |
|  | SOD-R | 5'-AGCCACACACCAGAAGATACACAC-3' |
|  | APX-F | 5'-CCTCCGGAGGGTATCGTTATCTA-3' |
|  | APX-R | 5'-ACAGCCAGAAACATTGTCCAAAAGG-3' |
|  | AREB1-F | 5' TTCATCACTCTCTCCGTCTCC 3' |
|  | AREB1-R | 5' TGTTTGGTCTGCCGTGAATA 3' |
|  | RD29A-F | 5'-GATTTCTTCTGATCGACAAAACCTA-3' |
|  | RD29A-R | 5'-AGCAAACCCAACTTATTACATTACG-3' |
|  | RD29B-F | 5'-GCAAGCAGAAGAACCAATCA-3' |
|  | RD29B-R | 5'-CTTTGGATGCTCCCTTCTCA-3' |
|  | RD22-F | 5’-AGGGCTGTTTCCACTGAGG-3’ |
|  | RD22-R | 5’-CACCACAGATTTATCGTCAGACA-3’ |
|  | RD20-F | 5'-TTAGCTCCGGTCACCAGTCA-3' |
|  | RD20-R | 5'-CATGTATGGTTTTGGTAATGTTTCC-3' |
|  | RAB18-F | 5’ –GGCTTGGGAGGAATGCTT-3’ |
|  | RAB18-R | 5’-TTGATCTTTTGTGTTATTCCCTTCT-3’ |
|  | KIN2-F | 5’-GCAACAGGCGGGAAAGAGTAT-3’ |
|  | KIN2-R | 5’-CCGGTCTTGTCCTTCACGAA-3’ |
